# Reasons for Composite Restoration Replacement in an Iranian Population: Composite Restoration Replacement in Patients

**DOI:** 10.31661/gmj.v13iSP1.3618

**Published:** 2024-12-29

**Authors:** Shima Nemati Jole Karan, Kowsar Nouroozi Loraki, Elham Ghanatir

**Affiliations:** ^1^ Department of Psychiatry, Golestan Hospital ,School of Medicine, Ahvaz Jundishapur University of Medical Sciences, Ahvaz, Iran; ^2^ Department of Restorative Dentistry, School of Dentistry, Ahvaz Jundishapur University of Medical Sciences, Ahvaz, Iran

**Keywords:** Composite Resins, Dental Restoration Failure, Dental Caries

## Abstract

**Background:**

This study aimed to assess the reasons for composite restoration replacement in patients in Ahwaz, Iran.

**Materials and Methods:**

This cross-sectional study was conducted on 231 patients presenting to the dental clinic of the School of Dentistry, Ahwaz Jundishapour University in 2017-2018 who required composite restoration replacement. A senior dental student performed a clinical dental examination of patients using a dental explorer, a dental mirror, and dental floss, and the decayed, missed, and filled (DMF) index of the patients was recorded. The patients also underwent radiography. The reason for composite restoration replacement was recorded. Data were analyzed by independent t-test, Chi-square test, one-way ANOVA, and LSD test (alpha=0.05).

**Results:**

Of 231 patients, 104 (45%) were males and 127 (55%) were females. The majority of the patients (27.3%) were between 40-50 years and had Class II malocclusion (62%). The mean DMF of patients was 3.48±1.36. Maxillary anterior teeth comprised the majority of the teeth that required restoration replacement. Secondary caries was the most common cause of restoration replacement (23.4%), followed by a combination of secondary caries and pain or dentin hypersensitivity (15.1%). The reason for restoration replacement had a significant association with age with primary caries being most prevalent in the 41-50 age group (P0.05) but had no significant association with gender or class of occlusion (P0.05). Patients requiring restoration replacement due to primary and secondary caries, and broken or cracked restorations had significantly higher mean DMF values compared to those with other reasons (P=0.031).

**Conclusion:**

Secondary caries was the most common reason for composite restoration replacement in the study population, and the reason for restoration replacement had a significant association with age and DMF of patients.

## Introduction

Dental composite resins are increasingly used for tooth-colored restorations due to their optimal esthetics, conservativeness, and ability to bond to tooth structure. They have largely replaced amalgam restorations worldwide [1][2]. However, composite resins have high technical sensitivity [3] and cannot ideally prevent marginal leakage [1].

The longevity of dental restorations depends on the type of restorative material, size of cavity [4], degree of polymerization of composite resin [5], expertise and experience of dental clinician [6], patient’s age [7], level of cooperation and oral hygiene of patient [2][5], number of restored tooth surfaces [8], tobacco use and cigarette smoking [9], age of restoration, and quality of isolation. Evidence shows that the main reasons for composite restoration replacement include secondary caries, marginal fracture of restoration, discoloration, tooth hypersensitivity, periapical abscess, or loss of anatomical contour of restoration [10]. Polymerization shrinkage results in gap formation at the tooth-composite interface and subsequent leakage of saliva and bacteria, leading to composite restoration failure [11].

Correct detection of the tooth-restoration interface is the main problem encountered in composite restoration replacement, which results in excessive removal and weakening of the remaining tooth structure [12]. A previous study reported that dental clinicians were responsible for restoration failure in 30% of the cases; patients and quality of dental restorative materials were responsible for 47% and 23% of the failures, respectively [13]. Poor knowledge of dental clinicians about the biological, chemical, and physical properties of the tooth structure results in early failure of restorations, compromises the integrity of the tooth structure, and results in recurrent caries and subsequent pulp necrosis, necessitating root canal therapy and prosthetic crown placement [14].

Restoration replacement is often associated with enlargement of the cavity size, weakening of the residual tooth structure and restoration, and further damage to both the tooth and restoration [15].

Information about the frequency of different reasons for restoration replacement can help in strategy planning to prevent or minimize future restoration failures. Considering the scarcity of studies in this respect in Iran, this study aimed to assess the reasons for composite restoration replacement in Ahwaz, Iran.

## Materials and Methods

**Table:** Table1. Main Reasons for Composite Restoration Replacement in the Present Study

**Reason**	**Males**		**Females**		**Total**		**P-value**
	**n**	**%**	**n**	**%**	**n**	**%**	
**Primary caries**	14	13.5	8	6.3	22	9.5	
**Secondary caries**	25	24	29	22.8	54	23.4	
**Defective margins**	0	0	3	2.4	3	1.3	
**Isthmus fracture**	1	1	1	0.8	2	0.9	
**Failed or cracked restoration **	1	1	0	0	1	0.4	
**Lost restoration**	5	4.8	9	7.1	14	6.1	
**Tooth fracture**	3	2.9	1	0.8	4	1.7	
**Open contact**	10	9.6	9	7.1	19	8.2	0.64
**Overhang**	3	2.9	6	4.7	9	3.9	
**Dentin hypersensitivity**	3	2.9	5	3.9	8	3.5	
**Other reasons**	12	11.5	16	12.6	28	12.1	
**Secondary caries + pain or hypersensitivity **	16	15.4	19	15	35	15.1	
**Secondary caries + other **	2	1.9	3	2.4	5	2.2	
**Secondary caries + broken or cracked restoration **	2	1.9	4	3.1	6	2.6	
**Other**	7	6.7	14	11	21	9.9	
**Total**	104	100	127	100	231	100	

This cross-sectional study was conducted on 231 patients presenting to the dental clinic of the School of Dentistry, Ahwaz Jundishapour University in 2017-2018 who required composite restoration replacement. The study protocol was approved by the ethics committee of the university and written informed consent was obtained from all patients prior to their enrollment. All participants were required to be at least 10 years old and to have at least one composite restoration that needed replacement. Patients were also required to provide written informed consent and to be willing to undergo a comprehensive clinical dental examination, including the use of a dental explorer, dental mirror, and dental floss, as well as radiographic imaging. Additionally, patients with a full set of permanent teeth (excluding third molars) and those who had no systemic conditions or medications that could affect dental health were included in the study.

### Sample Size

The sample size was calculated to be 231 patients assuming a 95% confidence interval, P=0.308, and d=0.06 using the sample size calculation formula.

### Data Collection

A senior dental student performed clinical dental examination for all patients using a dental explorer, a dental mirror, and dental floss under adequate lighting, and the decayed, missed, and filled (DMF) index of patients was recorded. Radiography was also performed.

The assessment of the DMF index involved a systematic clinical examination of an individual’s teeth to quantify the presence of dental caries, missing teeth due to caries, and teeth that had been restored. Each tooth was visually inspected and probed using standardized dental instruments to identify and record the specific condition of each tooth surface. The DMF index was calculated by summing the number of decayed (D), missing (M), and filled (F) teeth or surfaces, providing a comprehensive measure of the individual’s dental health status. For the DMFT index, which assesses the number of decayed, missing, and filled teeth, the score can range from 0 to 28 for an adult with a full set of permanent teeth (excluding third molars).

Age, gender, class of occlusion, type of tooth with failed/defective composite restoration, and restoration type were all recorded. Occlusion is classified into three main classes: Class I, where the molars are in a normal relationship with the upper first molar’s mesiobuccal cusp occluding in the buccal groove of the lower first molar; Class II, characterized by the upper first molar’s mesiobuccal cusp occluding anterior to the buccal groove of the lower first molar, often indicating an overbite; and Class III, where the upper first molar’s mesiobuccal cusp occludes posterior to the buccal groove of the lower first molar, often indicating an underbite.

The restorations were classified according to the G.V. Black classification system, which includes Class I through Class V restorations, as well as specific subcategories for Class II restorations (mesio-occlusal/disto-occlusal and mesio-occluso-distal).

The reason for restoration replacement was recorded based on the following definitions: Primary caries, defined as the presence of caries not related to the main restoration; secondary caries, characterized by caries beneath the restoration or at the restoration margins in direct contact with the restoration, detectable clinically with a dental explorer or on radiographs; defective restoration margins (without caries), indicating the presence of defective margins without the anatomical form and no occlusal contact with the opposing tooth; isthmus fracture, referring to the fracture of the restoration isthmus; broken or cracked restoration, involving a true fracture in the composite mass; lost restoration, a restoration lost due to poor retention without underlying caries; tooth fracture, a fracture of a part of the tooth structure in contact with the restoration due to weakening or undermining; open contact, the presence of symptoms such as food impaction and easy passage of dental floss suggesting an open contact; overhang, confirmed by the sharp tip of an explorer and radiographic evidence; pain or hypersensitivity, pain or discomfort in response to thermal stimuli; and other reasons, encompassing any other issues not falling into the above categories.

### Statistical Analysis

Data were analyzed by SPSS version 24 (SPSS Inc., IL, USA) using independent t-test, Chi-square test, one-way ANOVA, and LSD test at 0.05 level of significance.

## Results

**Table:** Table2. Reasons for Restoration Replacement based on the Age Group (yrs.) of Patients

**Reason**	**10 to 20**		**21-30**		**31- 40**		**41-50**		**51-60**		**61-70**		**Total**		**P-value**
	**n**	**%**	**n**	**%**	**n**	**%**	**n**	**%**	**n**	**%**	**n**	**%**	**n**	**%**	
**Primary caries**	0	0	1	1.8	4	6.7	12	19	5	15.2	0	0	22	9.5	
**Secondary caries**	2	25	7	12.3	14	23.3	17	27	13	39.4	1	10	54	23.4	
**Defective margins**	0	0	3	5.3	0	0	0	0	0	0	0	0	3	1.3	
**Isthmus fracture**	0	0	2	3.5	0	0	0	0	0	0	0	0	2	0.9	
**Failed or cracked restoration **	0	0	0	0	0	0	0	0	1	3	0	0	1	0.4	
**Lost restoration**	1	12.5	5	8.8	6	10	0	0	1	3	1	10	14	6.1	
**Tooth fracture**	0	0	3	5.3	0	0	1	1.6	0	0	0	0	4	1.7	
**Open contact**	0	0	9	15.8	5	8.3	3	4.8	0	0	2	20	19	8.2	
**Overhang**	0	0	4	7	1	1.7	2	3.2	1	3	1	10	9	3.9	**0.007
**Dentin hypersensitivity**	0	0	3	5.3	2	3.3	2	3.2	1	3	0	0	8	3.5	
**Other reasons**	1	0	7	12.3	10	16.7	8	12.7	1	3	1	10	28	12.1	
**Secondary caries + pain or hypersensitivity **	1	12.5	7	12.3	12	20	8	12.7	7	21.2	0	0	35	15.1	
**Secondary caries + other **	1	12.5	0	0	2	3.3	1	1.6	1	3	0	0	5	2.2	
**Secondary caries + broken or cracked restoration **	0	0	2	3.5	0	0	2	3.2	0	0	2	20	6	2.6	
**Other**	2	25	4	7	4	6.7	7	11.1	2	6.1	2	20	21	9.9	
**Total**	8	100	57	100	60	100	63	100	33	100	10	100	231	100	

**Table:** Table3. Reasons for Restoration Replacement based on the Class of Occlusion

**Reason**	**Class I**		**Class II**		**Class III**		**Total**		**P-value**
	**n**	**%**	**n**	**%**	**n**	**%**	**n**	**%**	
**Primary caries**	2	4.5	17	11.9	3	6.8	22	9.5	0.144
**Secondary caries**	8	18.2	37	25.9	9	20.5	54	23.4	
**Defective margins**	0	0	3	2.1	0	0	3	1.3	
**Isthmus fracture**	0	0	2	1.2	0	0	2	0.9	
**Failed or cracked restoration **	0	0	1	0.7	0	0	1	0.4	
**Lost restoration**	5	11.4	7	4.9	2	4.5	14	6.1	
**Tooth fracture**	0	0	3	2.1	1	2.3	4	1.7	
**Open contact**	3	6.8	12	8.4	4	9.1	19	8.2	
**Overhang**	0	0	8	5.6	1	2.3	9	3.9	
**Dentin hypersensitivity**	2	4.5	6	4.2	0	0	8	3.5	
**Other reasons**	4	9.1	12	8.4	12	27.3	28	12.1	
**Secondary caries + pain or hypersensitivity **	10	22.7	17	11.9	8	18.2	35	15.1	
**Secondary caries + other **	3	3.8	2	1.4	0	0	5	2.2	
**Secondary caries + broken or cracked restoration **	2	4.5	3	2.1	1	2.3	6	2.6	
**Other**	5	11.4	3	9.1	3	6.8	21	9.9	
**Total**	44	100	143	100	44	100	231	100	

A total of 231 patients were evaluated including 104 males (45%) and 127 females (55%).

Table-1 presents the main reasons for composite restoration replacement in the present study. As shown, secondary caries was the most common cause of restoration replacement (23.4%) followed by a combination of secondary caries and pain or dentin hypersensitivity (15.1%) in general, and also separately in males and females. The Chi-square test showed no significant difference between males and females in reasons for restoration replacement (P=0.640).

Of all patients, 63 (27.3%) were between 41-50 years, 60 (26%) were between 31-40 years, 57 (24.7%) were between 21-30 years, 33 (14.3%) were between 51-60 years, 10 (4.3%) were between 61-70 years, and 8 (3.5%) were between 10-20 years. Of all teeth that required restoration replacement, 71.4% (n=165) were maxillary anterior teeth (highest frequency), 13.4% (n=31) were maxillary premolars, 6.9% (n=16) were mandibular premolars, 4.8% (n=11) were mandibular anterior teeth, 3% (n=7) were maxillary molars, and 0.4% (n=1) were mandibular molars.

Class III restorations had the highest frequency (40.7%, n=94) followed by Class IV (29.9%, n=69), Class II-mesio-occlusal/disto-occlusal (13.4%, n=31), Class V (10%, n=23), Class I (3.9%, n=9), and Class II mesio-occluso-distal restorations (2.2%, n=5).

Secondary caries was most common in the proximal surfaces (66.7%, n=92) followed by the occlusal surface (22.5%, n=31) and cervical region (10.9%, n=15).

Table-2 presents the reasons for restoration replacement based on the age group of patients. The Chi-square test showed a significant difference among different age groups in reasons for restoration replacement (P=0.007). Secondary caries were the main reason for restoration replacement in 10-20-, 31-40-, 41-50-, and 51-60-year-old age groups. Open contact of restoration was the main reason for restoration replacement in 21-30-, and 61-70-year-olds. The highest frequency of restoration replacement was recorded in 21-to 50-year-olds, which was due to secondary caries.

Of all, 62% (n=143) were Class II, 19% (n=44) were Class I, and 19% (n=44) were Class III. Table-3 presents the reasons for restoration replacement based on the class of occlusion. The main reason for restoration replacement was secondary caries plus pain or dentin hypersensitivity in Class I (22.7%), secondary caries alone in Class II (25.9%), and other reasons in Class III (27.3%) cases. The Chi-square test showed no significant difference in reasons for restoration replacement among different classes of occlusion (P=0.144). The mean DMF of patients was 3.48±1.36 (range 0.67 to 8.67). Table-4 shows the reasons for restoration replacement based on the DMF of patients. One-way ANOVA revealed a significant difference in reasons for restoration replacement according to the mean DMF (F=1.90, P=0.031). Pairwise comparisons by the LSD test showed that in patients requiring restoration replacement due to primary caries, the mean DMF was significantly higher than that in patients requiring restoration replacement due to defective margins (without caries), isthmus fracture, lost restoration, open contact, and other reasons (P<0.05). In patients requiring restoration replacement due to secondary caries, the mean DMF was significantly higher than that in patients requiring restoration replacement due to defective margins (without caries) and isthmus fracture (P<0.05). In patients requiring restoration replacement due to defective margins (without caries), the mean DMF was significantly lower than that in patients requiring restoration replacement due to secondary caries plus pain or dentin hypersensitivity, and secondary caries plus other reasons (P<0.05). In patients requiring restoration replacement due to isthmus fracture, the mean DMF was significantly lower than that in patients requiring restoration replacement due to secondary caries plus pain or dentin hypersensitivity, secondary caries plus other reasons, secondary caries plus broken or cracked restoration, and other reasons (P<0.05). Also, patients who required restoration replacement due to primary and secondary caries, and broken or cracked restoration had a higher mean DMF than others.

## Discussion

**Table:** Table4. Reasons for Restoration Replacement based on DMF of Patients

**Reason**	**n**	**DMF (M ± SD)**
**Primary caries**	22	4.09 ± 1.27
**Secondary caries**	54	3.76 ± 1.44
**Defective margins**	3	2.00 ± 0.33
**Isthmus fracture**	2	1.33 ± 0.0
**Failed or cracked restoration **	1	4.33 ± 0.0
**Lost restoration**	14	2.88 ± 1.03
**Tooth fracture**	4	2.92 ± 1.2
**Open contact**	19	3.07 ± 1.21
**Overhang**	9	3.15 ± 1.25
**Dentin hypersensitivity**	8	3.29 ± 1.22
**Other reasons**	28	3.19 ± 1.4
**Secondary caries + pain or hypersensitivity **	35	3.65 ± 1.29
**Secondary caries + other **	5	4.07 ± 1.46
**Secondary caries + broken or cracked restoration **	6	3.67 ± 1.17
**Other**	21	3.54 ± 1.41
**Total**	231	3.48 ± 1.36

This study assessed the frequency of reasons for composite restoration replacement in Ahwaz, Iran. The results showed that secondary caries was the most common reason for restoration replacement (23.4%), followed by a combination of secondary caries and pain or dentin hypersensitivity (15.1%). Chrysanthakopoulos [16] reported that primary caries (60%) was the main cause of composite restoration of teeth while secondary caries (48%) was the most common reason for composite restoration replacement, which was in agreement with the present results. Discoloration was the second most common reason for restoration replacement in their study. Abolghasemzade et al. [17] demonstrated that secondary caries were the most common reason for composite restoration replacement in Babol, Iran in 2013 and 2014. The same results were reported by Braga et al, [18] Frost [19], and many others [20][21][22][23][24]. Soares and Cavalheiro [15] reported that secondary caries were the reason for the replacement of 41% of amalgam and 36% of composite restorations in Portugal. Composite fracture, caries, marginal defects, and lack of proximal contact (open contact) were reported as the main reasons for composite restoration replacement by Opdam et al [25]. In a review study, Deligergi et al. [26] concluded that clinically evident secondary caries was the main reason for the replacement of dental restorations. Similar results were reported by many other studies [7][27][28][29]. The high incidence of secondary caries under composite restorations can be explained by microbiological findings [30]. The high technical sensitivity of composite restorations also contributes to the occurrence of secondary caries. Moreover, the final clinical outcome of composite restorations highly depends on the oral hygiene status of patients. Composite restorations can enhance the proliferation of Streptococcus mutans as the main microorganism responsible for dental caries. This parameter combined with poor oral hygiene can lead to the occurrence of secondary caries [31]. A high load of Streptococcus mutans has been reported at the composite restoration margins compared with amalgam and glass ionomer restoration margins [32]. Some other studies reported higher dental plaque accumulation at the tooth-composite interface compared with the tooth-amalgam interface [33] and confirmed that resin materials result in greater accumulation of dental plaque with a more cariogenic composition compared with amalgam, silicate cement, and glass ionomer [16]. Composite resin shrinkage is another factor contributing to the occurrence of secondary caries. Polymerization shrinkage of resin materials results in gap formation at the tooth-restoration interface, particularly at the gingival margin of the tooth [6].

Therefore, measures must be taken to minimize polymerization shrinkage of composite resins [16]. Microleakage is another parameter related to the development of secondary caries [34]. A previous study reported that a tooth-restoration interface gap>35-50 µm can predispose the tooth to secondary caries [35]; while some other studies found no significant correlation between gap size and secondary caries development except in case of macro-leakage (gaps>250-400 µm) [34]. It appears that secondary caries do not develop due to microleakage at the tooth-restoration interface; it is smooth-surface caries that develop due to reasons similar to those of primary caries [6].

A 10-year study carried out by Gaengler et al. [36] reported that restoration fracture was the most common cause of restoration replacement in the first 5 years while secondary caries was the main cause of replacement in the second 5 years. However, Raskin et al, [37] and Mair [38] reported very few or no cases of secondary caries as the reason for restoration replacement, which may be attributed to the routine use of rubber dam isolation for restorative procedures in European countries, which results in optimal isolation and minimizes the risk of caries recurrence.

Aside from secondary caries, composite restoration replacement may be required due to other reasons such as restoration fracture, marginal defects, tooth fracture, marginal discoloration, poor anatomical contour, and over-contouring of restorations [23][26][39][40]. As mentioned earlier, the success of restorations depends on a number of factors such as quality of restorative material, size and type of restoration, tooth type, experience and expertise of dental clinician, tooth location in the dental arch, number of restored tooth surfaces, and patient’s age [17].

In the present study, the Class of occlusion had no significant association with the reason for restoration replacement. However, Class III and IV composite restorations had a higher frequency of failure. Similar results were reported by Nikaido et al [41]. A previous study reported that the 5-year survival rate of Class III, IV, and V restorations was 54.6%, 47.7%, and 59.2%, respectively [42]. A higher frequency of failure in Class III and IV composite restorations can be due to poor color match, discoloration, or caries, the significance of the optimal appearance of restorations in the esthetic zone, and higher demand of patients for their replacement. It should be noted that the gingival regions in Class II, III, IV, and V restorations are susceptible to secondary caries. The reason may be more difficult clinical control and oral hygiene maintenance at the gingival margins and cervical regions [17][40]. This statement explains the higher rate of restoration replacement in Class II, III, IV, and V cavities compared with Class I and VI restorations [40]. Abolghasemzade et al. [17] reported that Class II restorations were the most common restoration type requiring replacement, accounting for over 50% of the cases. They explained the reason for be difficult restoration of posterior teeth. This result was different from the present findings, which may be due to the small number of Class II posterior composite restorations in the present study. In the current study, maxillary anterior teeth comprised the majority of the teeth that required restoration replacement, which was in line with previous findings [17] and may be due to the higher frequency of use of composite resin for anterior teeth, compared with posterior teeth, as a result of esthetic considerations and higher cost. Also, problems such as coronal and marginal discoloration and caries are more easily detected by patients with anterior teeth. Therefore, replacement of anterior composite restorations is a major complaint of many patients [17].

Assessment of the correlation of reasons for restoration replacement and demographic factors revealed no significant difference in this regard between males and females, which was in line with some [6][40], and in contrast to some other studies [23]. Differences in the results in this respect may be due to differences in sample size and study populations. Nonetheless, reasons for restoration replacement had a significant correlation with the age group of patients. Patients between 20-50 years had the highest frequency of restoration replacement, which was mainly due to secondary caries. Higher occupational involvement in this age group probably leads to less attention to oral hygiene and the occurrence of secondary caries. The frequency of restoration replacement and secondary caries was lower in patients between 50-70 years probably due to tooth extraction, less popularity of composite restorations in the past, and lower frequency of dental visits in this age group.

The present results revealed a significant correlation between reasons for restoration replacement and DMF of patients, and patients who required restoration replacement due to primary or secondary caries, and broken or cracked restoration had a higher DMF, which is justifiable by the fact that higher DMF is associated with a higher frequency of carious, extracted, and filled teeth and poorer oral hygiene.

The relatively small sample size was a limitation of this study. Future studies with a larger sample size are required to compare the reasons for composite and amalgam restoration replacements. Also, the success rate of composite restorations performed by general dentists and restorative dentists should be compared.

## Conclusion

In conclusion, our study on composite restoration replacement in an Iranian population shows the significant role of secondary caries as the primary reason for restoration failure, followed by a combination of secondary caries and pain or dentin hypersensitivity. The findings indicate that patients in the 40-50 age group are particularly at risk for primary caries. The lack of significant association between restoration replacement and factors such as gender and occlusion class implies that these variables may not be major contributors to restoration failure in this population. Furthermore, the higher mean DMF values observed in patients requiring restoration replacement due to primary and secondary caries, as well as broken or cracked restorations, show the need for early intervention and maintenance to prevent the progression of dental diseases. These results emphasize the importance of regular dental check-ups, effective caries management, and the use of durable restorative materials to reduce the incidence of restoration replacement and improve long-term oral health outcomes.

## Conflict of Interest

The authors declare no competing interests.
